# Dr. C. M. Richmond’s Papers on Crown- and Bridge-Work

**Published:** 1892-06

**Authors:** 


### DR. C. M. RICHMOND'S PAPERS ON CROWN- AND BRIDGE-WORK.

### IMPORTANT NOTICE.

We will begin in the July number of this journal the publication of a series of illustrated articles by Dr. Richmond. They will extend through the year, and probably beyond, and will appear in each number until completed. They will include his entire experience in the preparation of crown- and bridge-work, which, with the illustrations prepared directly under his supervision, must prove of exceptional value to all engaged in this work.

Dr. Richmond's recent introduction of a removable bridge of his own devising has received the highest encomiums from those most competent to judge, and we feel sure that this process must eventually commend itself to the profession generally.

It was the intention to have had this series of articles appear at an earlier date, but, in order that our readers and all interested should have the opportunity to secure them, it was decided to commence their publication in July. This will enable new subscribers to begin at the regular subscription period.

